# Quality assurance of hyperspectral imaging systems for neural network supported plant phenotyping

**DOI:** 10.1186/s13007-024-01315-y

**Published:** 2024-12-19

**Authors:** Justus Detring, Abel Barreto, Anne-Katrin Mahlein, Stefan Paulus

**Affiliations:** Institute of Sugar Beet Research, Göttingen, Niedersachsen 37079 Germany

**Keywords:** Image resolution, Image sharpness, Spectral accuracy, Spatial accuracy, Illumination, Machine learning, Remote sensing, Plant diseases, Computer vision

## Abstract

**Background:**

This research proposes an easy to apply quality assurance pipeline for hyperspectral imaging (HSI) systems used for plant phenotyping. Furthermore, a concept for the analysis of quality assured hyperspectral images to investigate plant disease progress is proposed. The quality assurance was applied to a handheld line scanning HSI-system consisting of evaluating spatial and spectral quality parameters as well as the integrated illumination. To test the spatial accuracy at different working distances, the sine-wave-based spatial frequency response (s-SFR) was analysed. The spectral accuracy was assessed by calculating the correlation of calibration-material measurements between the HSI-system and a non-imaging spectrometer. Additionally, different illumination systems were evaluated by analysing the spectral response of sugar beet canopies. As a use case, time series HSI measurements of sugar beet plants infested with Cercospora leaf spot (CLS) were performed to estimate the disease severity using convolutional neural network (CNN) supported data analysis.

**Results:**

The measurements of the calibration material were highly correlated with those of the non-imaging spectrometer (r>0.99). The resolution limit was narrowly missed at each of the tested working distances. Slight sharpness differences within individual images could be detected. The use of the integrated LED illumination for HSI can cause a distortion of the spectral response at 677*nm* and 752*nm*. The performance for CLS diseased pixel detection of the established CNN was sufficient to estimate a reliable disease severity progression from quality assured hyperspectral measurements with external illumination.

**Conclusion:**

The quality assurance pipeline was successfully applied to evaluate a handheld HSI-system. The s-SFR analysis is a valuable method for assessing the spatial accuracy of HSI-systems. Comparing measurements between HSI-systems and a non-imaging spectrometer can provide reliable results on the spectral accuracy of the tested system. This research emphasizes the importance of evenly distributed diffuse illumination for HSI. Although the tested system showed shortcomings in image resolution, sharpness, and illumination, the high spectral accuracy of the tested HSI-system, supported by external illumination, enabled the establishment of a neural network-based concept to determine the severity and progression of CLS. The data driven quality assurance pipeline can be easily applied to any other HSI-system to ensure high quality HSI.

## Background

### Hyperspectral imaging systems

Hyperspectral imaging (HSI) originated in 1986 when the first airborne hyperspectral spectrometer for mineral mapping was launched by GER Corp. This was soon followed by a more advanced HSI-systems from NASA/JPL capable of collecting images in the range of 400 to 2500*nm* [1]. From this on, the development of various spectral sensors increased. Today, HSI has a wide variety of applications ranging from medicine, food safety, environment, geology and agriculture [2–6]. The potential of hyperspectral sensors to remotely measure spectral characteristics of objects is based on the fact that materials emit electromagnetic energy in signatures that correspond to their chemical composition and physical structure [7]. The emitted electromagnetic energy is considered as reflected light, which forms certain characteristic spectral signatures depending on the sensed object and its state. The advantage of HSI-systems compared to non-imaging systems is the acquisition of spatial information in the form of pixels. Each of these pixels contains the spectral information according to the spectral and spatial resolution of the HSI-system used.

In general, HSI-systems are categorized into four different types: push broom or rather line scanner, the filter-based systems, snapshot systems, and whisk broom systems [8]. One of the most established systems is the line scanner, which acquires images line by line. This approach implies that either the object or the system must be moved to enable a spatial measurement [9]. Currently available handheld line scanning systems are mirror based to enable line scanning [10], allowing flexible application for different use cases [11–14].

### HSI quality aspects for plant phenotyping

The introduction of high-end technologies such as HSI has greatly improved plant phenotyping. In general, plant phenotyping describes the response of a plant with its specific genetic background to various environmental factors and vice versa [15]. Especially in the field of plant disease detection, HSI has not only improved variety screening [16, 17], but also promoted precision agriculture by supporting decision making for crop protection, yield cataloging and fertilization [18–20]. Fungal plant diseases have become highly investigated objects for HSI in phytopathology [18, 21, 22] because of their strong influence on yield and food quality. There are multiple studies investigating Cercospora leaf spot (CLS) with HSI on different scales [11, 21, 23]. CLS causes distinct leaf spots that can be accurately localized in the spatial information of a hyperspectral image [11, 21].

In order to draw meaningful conclusions from the analysis of hyperspectral images, it is essential to increase the image quality as much as possible. The quality is characterised by several technical specifications, such as the spectral or the spatial resolution. Modern non-imaging hyperspectral sensors can measure in a spectral range between 350-2500*nm* of the electromagnetic spectrum [6]. This covers the visible (VIS, 400-750*nm*), near infrared (NIR, 750-1000*nm*), shortwave (SWIR, 1000-2500*nm*), and partially the ultraviolet (UV, 100-400*nm*) portion of the electromagnetic spectrum. The measurement of the spectral range is divided by spectral wavebands. The proximity and width of these wavebands define the spectral resolution of a given system, which can be less than 1*nm* in high resolution non-imaging spectrometers. Nevertheless, the correlation between wavebands situated in close proximity enables the measurement of systems with reduced spectral resolution, a common occurrence in the case of HSI-systems. [6]. To exploit the full sensitivity of a hyperspectral sensor and obtain the best possible image quality, it is important to consider additional factors before and during the measurement, such as illumination, spatial resolution, and image sharpness. Since the sensor measures the reflected energy originated from the illumination, spectral range and intensity of the illumination source have a significant impact on the image and data quality. In-field illumination conditions with sunlight as the source of illumination can change within seconds. Therefore, it is essential to use a reference material within each measurement.

The standard illumination for laboratory HSI setups are currently halogen lamps [8, 24]. Halogen lamps have high energy output in the VIS and NIR spectra [24] which are spectral regions covered by most HSI-systems. Light-emitting diodes (LEDs) have recently been introduced to HSI as a supplement to halogen lamps in the UV-blue region [25] and also as exclusive illumination [24, 26]. The strong light emission in the NIR spectral region of halogen lamps is beneficial for various plant phenotyping applications, since its considered as an important spectral region for plant spectroscopy. The light emission in the UV and VIS-blue region of halogen lamps may be insufficient depending on the application and HSI-system, where LEDs may be more convincing. In plant phenotyping, both illumination systems have to cope with the geometric structure of leaves and plants for HSI. Depending on the position of the illumination and its angle of incidence, the illumination may not be evenly distributed on the leaf or plant of interest. In addition, measurement angle and distance have a strong influence on data quality and the level of detail [12, 27]. This is one of the major challenges in HSI for plant phenotyping.

Spatial accuracy of hyperspectral images depend on the spatial resolution and image sharpness. Line scanning spectral sensors have a fixed line length defined by a certain number of pixels, which determines the spatial dimension of the y-axis of the image. The length of the x-axis is variable and depends on the movement distance of either the system or the object of interest. To apply line scanning HSI in uncontrolled conditions, devices have been developed with a fixed spatial dimension where neither the HSI-system nor the object has to be moved for measuring. Handheld line scanning HSI-systems with a fixed focal length, have limitations in varying the measurement distance when a sharp hyperspectral image is desired. The focal length combined with the sensor chip size defines the field of view. The focal ratio which is describing the light gathering ability is influencing the sharpness of an image in relation to the distance of the measured object as well. In this context the plant geometry is once more challenging. Since plants are three dimensional structures not every latitude in the z-dimension can be measured with the maximum sharpness of an HSI-system. The sine-wave based spatial frequency response (s-SFR) is the international standard method for evaluating image resolution and sharpness of digital cameras [28]. This method is based on the Nyquist-Shannon sampling theorem [29], which has not been applied to hyperspectral images before in literature. There is always a trade-off between latitude focus, depth of field, and region of interest in relation to the field of view and measuring distance. Handheld line scanning HSI-systems, in particular, face difficulties in meeting these requirements. Therefore, it is essential to validate every newly developed HSI-system for its use in plant phenotyping.

### Machine learning in hyperspectral imaging driven plant phenotyping

The use of HSI in plant phenotyping produces complex and vast datasets that are challenging to handle. Machine learning, a subfield of artificial intelligence, allows for in-depth analysis of HSI data. Classical regression models typically rely on one-dimensional parameters. For example, if the model is designed to classify a pixel in an image based on its spectral characteristics, it only takes into account individual spectral values within the measured spectral range. More advanced machine learning methods, such as neural networks, can take the entire complexity of spectral signatures of the pixel’s spectrum into consideration for classification [30]. In addition, neural networks are capable of recognizing the morphology of objects in images, expanding data analysis to include spatial information. Neural networks are favourable for pattern recognition, which enables precise understanding of HSI data and improves the extraction of plant traits. In particular, convolutional neural networks (CNN) are predestined for image analysis. CNN’s main architectures consist of several convolutional layers in which the data is analyzed, causing a reduction of unnecessary parameters and creating a holistic understanding on the analyzed data points [31]. Liu et al. [30] designed a CNN architecture specifically for analyzing spectral data. The designed CNN architecture leads to a one-dimensional analysis of the whole spectrum, making it highly applicable for multiclass pixel classification based on HSI data.

**Fig. 1:**
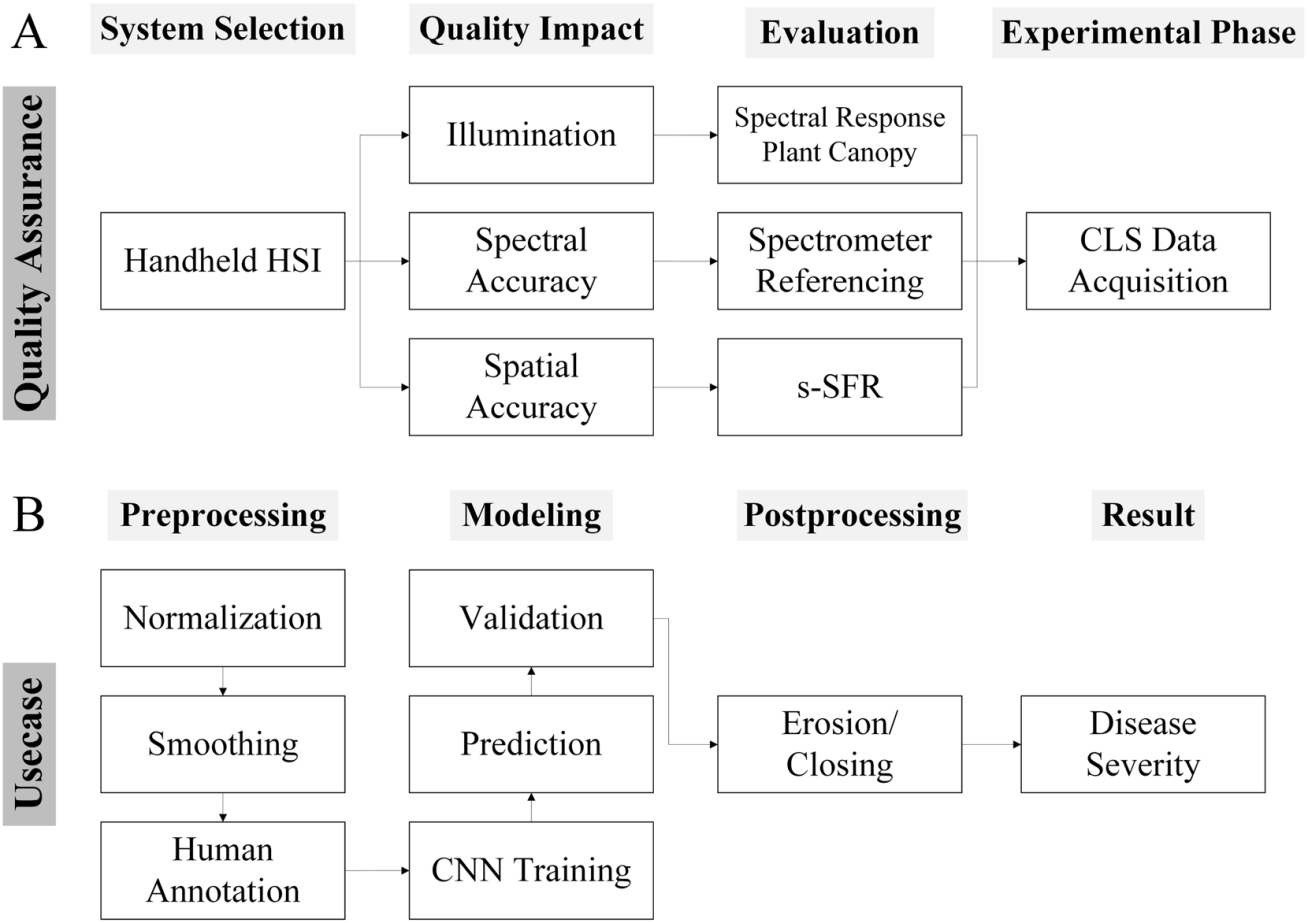
Quality assurance pipeline of hyperspectral imaging (HSI) systems with a convolutional neural network (CNN) supported data analysis concept. In Fig. 1** A** an evaluation of three crucial parameters is presented. The spatial accuracy was tested by analyzing the sine-wave based spatial frequency response (s-SFR). To investigate the artificial illumination, spectral responses of plant canopy’s were compared. The spectral accuracy of the HSI-system is validated by compering measurements of a calibration material with a spectrometer. After evaluating the parameters and adapting the measurements according to the results, the HSI-system is used to measure the disease progression of Cercospora leaf spot (CLS) infected sugar beet plants. In Fig 1** B** the concept for CNN supported HSI data analysis is presented. In the first step common spectral and image preprocessing steps such as normalization and smoothing are conducted. For supervised machine learning, the training data set has to be humanly annotated to define certain classes of interest. After training the model with humanly annotated data, the model can predict the classes for the whole dataset, which can be validated by comparing the results with humanly annotated data which has been excluded from the training process. To enhance the models’ performance, image postprocessing steps such as erosion and closing have been applied. After postprocessing the CLS disease severity has been derived from the model output

To accomplish high accuracys for such classifications high quality hyperspectral images are necessary. Following a pipeline is proposed (Fig. 1) to evaluate critical quality aspects of hyperspectral images such as spatial and spectral accuracy and illumination systems for HSI. Furthermore, a usecase is presented to analyse quality assured hyperspectral images supported by neural networks for phenotyping of plant disease severity progression.

## Methods

### Hyperspectral imaging system technical aspects

**Fig. 2:**
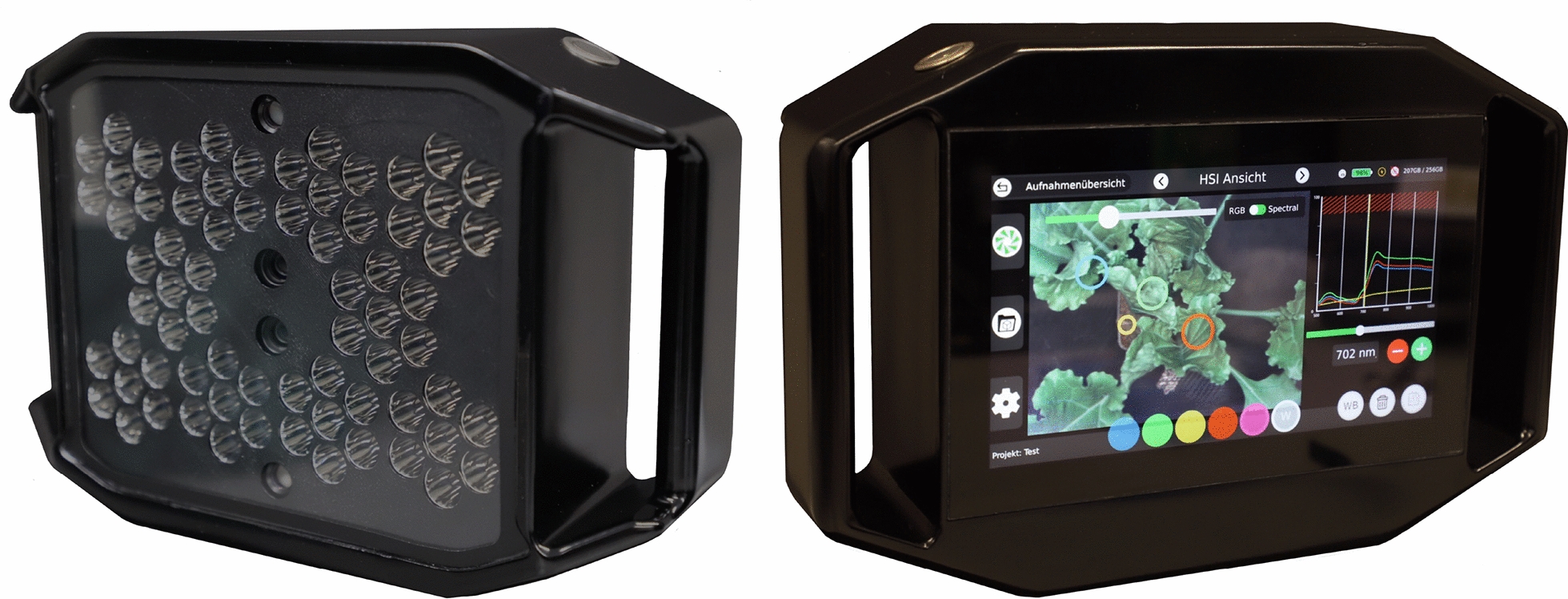
Blackmobile front and back view. The frontside shows a 7” LED touchscreen that displays the user interface running the hypercube view widget. The backside shows the broadband VIS/NIR LED array, the lenses of the hyperspectral and RGB camera, and the laser system for vertical alignment

A handheld hyperspectral line scanning imaging system (Blackmobile, HAIP Solutions GmbH, Hannover, Germany; Fig. 2; hereafter: HC) was used to proof and demonstrate the proposed pipeline to ensure high quality HSI measurements for plant phenotyping.

The HC is equipped with a metal-oxide-semiconductor (CMOS) VNIR hyperspectral sensor and an ultra high resolution (4K) RGB sensor (Tab. 1). The built-in CMOS sensor is capable of measuring incident electromagnetic energy between 500-1000*nm* with a spectral resolution of 5*nm*, resulting in 100 spectral bands per measurement (z-dimension). The spatial resolution of the image outcome consists of 640*480*px* (x,y-dimension). The exposure time for the line scan procedure can be adjusted between 1000-5000${\mu }s$ per line. A gain function is provided to adjust the sensitivity of the sensor by increasing or decreasing the current supply of the sensor. The optimal working distance of 50*cm* between the object and the lenses can be assigned by a laser system that calculates the distance between the object and the lenses by a trigonometric measurement. A novelty in handheld HSI-systems is the integrated illumination consisting of 70 broadband VIS/NIR high-power LEDs mounted on the back of the HC next to the sensor lenses (Fig. 2). Further technical details of the HC and the installed sensors are given in Table 1.

**Table 1:** HAIP Blackmobile technical details sensors

Parameter	HSI	RGB
Sensor	CMOS	CMOS
Sensor size	5568*3132${\mu }m$	7680*4320${\mu }m$
Sensor resolution	2 Megapixel	8 Megapixel
Pixel size	2.9*2.9${\mu }m$	2.0*2.0${\mu }m$
Focal ratio	1.8	1.8
Focal length	12*mm*	12*mm*
Spectral range	500-1000*nm*	NA
Spectral resolution	5*nm*	NA
Spectral bands	100	NA
Exposure	1000-5000${\mu }s$	Auto-Exposure/0.1-33.3*s*
Gain (analog)	1-15.5 (Multiplier)	1-15.5 (Multiplier)
Image resolution	640*480*px*	3840*2160*px*
Data depth	10*bit*	8*bit*
Peak signal-to-noise ratio	40.8*dB*	39*dB*
Working distance	45-55*cm*	45-55*cm*
FOV at 50*cm* distance	22*16.5*cm*	33*18*cm*
Data format	ENVI	JPG

The HC is operated via a 7” full high-definition (HD) LED touch screen and two physical buttons for booting the system and triggering the measurement. Besides setting options, such as exposure and gain, the user interface includes processing functions for the acquired data. By measuring an image-filling reference material, this measurement can be set as a “global reference” for pixel-by-pixel normalizing of the hyperspectral image. If the reference material is placed within the measurement of an object of interest, a round area of interest marker can be set at that location and adjustable in size to match the size of the reference material. By selecting this marker as the “global reference,” an average spectrum of the adjusted area of the marker is calculated and set as the global reference value used for normalizing of each pixel in the selected hyperspectral image. Both normalization methods calculate the reflectance following the formula.

$$reflectance = \frac{HSI - sensor dark current }{reference HSI - sensor dark current}$$

Due to technical variability such as heat or voltage, the noise produced by hyperspectral sensors varies with each measurement. To eliminate these differences during the normalization process, a dark current is recorded with each measurement and subtracted from the HSI, respectively. The normalized hyperspectral image can be examined in the widget, and multiple markers of interest can be set in the widget to display the average spectrum of the set area. The acquired data is saved as an ENVI formatted hyperspectral cube and the RGB image as a JPG image, which is automatically recorded before the spectral measurement starts, if enabled. Further technical details of the camera and its operating software are given in Table 2.

**Table 2:** HAIP Blackmobile technical details of the operating software and system hardware

Parameter	Value
User interface	HAIP Blackmobile Software
Operation System	L4T 32.5 - Ubuntu 18.04 - Linux kernel 4.9
Embedded Computer	NVIDIA$\circledR$ $\text {Jetson Nano}^{\text {TM}}$
CPU	Quad-Core ARM$\circledR$ Cortex$\circledR$ - A57 MP core
GPU	$\text {NVIDIA Maxwell}^{\text {TM}}$, 128 NVIDIA CUDA$\circledR$
RAM	4 GB 64-bit LPDDR4
Storage integrated	16*GB* eMMC 5.1-flash storage
Storage external	256*GB* SD
Battery	Li-Ion 14.4 V
Operational time	100 measurements
Display and operating unit	7” LED touch screen (full HD) and 2 buttons
Serial Connection and power socket	USB type-c
WIFI	2.4 GHz IEEE 802.11
Supply voltage	20*VDC*
Size (L*W*H)	250*165*70*mm*
Weight	1.5*kg*
Operational temperature	$10-30^{\circ }$C
Integrated illumination	Broadband VIS/NIR LED array
LED quantity	70

### Measuring setup

**Fig. 3:**
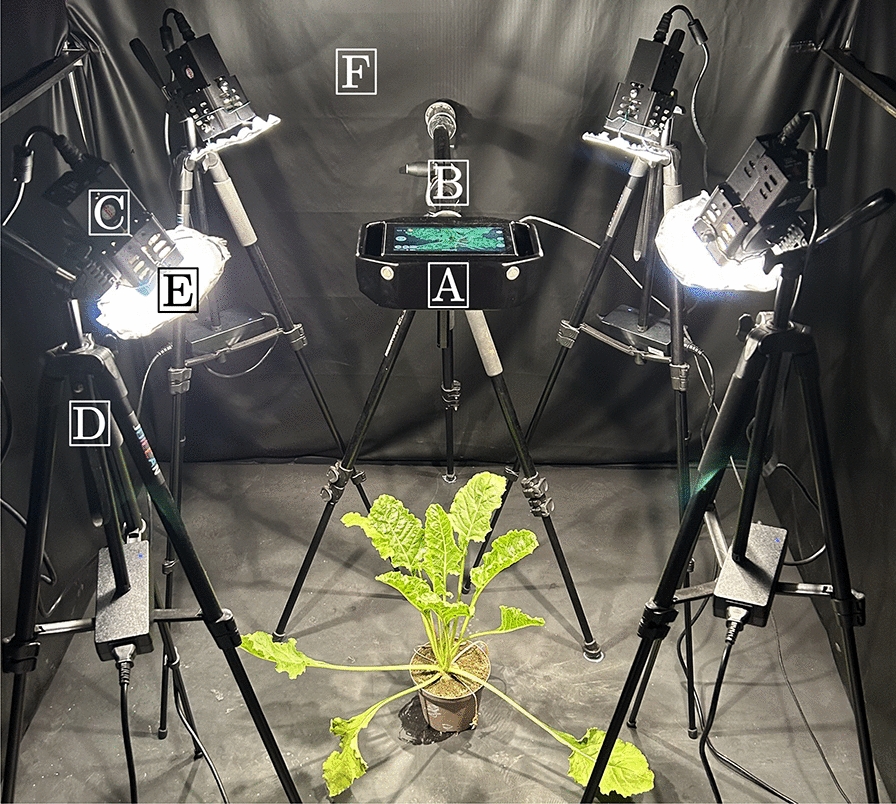
Measurement setup for hyperspectral imaging.** A**: HC,** B**: Tripod HC (height: about 80*cm*),** C**: External illumination,** D**: Tripod illumination (height: 108*cm*, angle: $40^{\circ }$),** E**: Diffusing screen,** F**: Blacked out measurement chamber (dimensions: 1.5*1.5*2*m* (L*W*H))

The framework of the measurement chamber is made of aluminum profiles and has a dimension of 1.5*1.5*2*m* (L*W*H). Light interference in the measurement chamber was prevented by blackening the walls with polyurethane-coated black nylon fabric (Blackout Fabric, Thorlabs Inc., Newton, United States), the floor was covered with a matte black lacquered wooden panel. The ceiling was left open to ensure adequate ventilation and to prevent heat stress to the plants (Fig. 3).

The external halogen illumination consisted of four 70*watt* voltage stabilized quartz tungsten halogen lamps (Illuminator Lamp, Malvern Instruments, Malvern, United Kingdom). To ensure evenly distributed illumination, round diffusing screens were fixed to the lamps with metal wires at a distance of 2*cm*. (nylon silk with an approximate light reduction of 1.0 f-stop). The lamps were mounted on 137*cm* tripods at an angle of about $40^{\circ }$. The tripods were adjusted to a height of 108*cm* above the ground and placed in a rectangle around the centre in a measurement chamber. The HC was mounted on an additional tripod. An barium sulfide plate with a dimension of 43*43*cm* (Specim Spectral Imaging Ltd., Oulu, Finland) was used as an image filling white reference material for all measurements.

### Plant material

Two sugar beet varieties were chosen for the experiments. BTS 8750 (Betaseed GmbH, Frankfurt, Germany) for investigating the quality of the hyperspectral illumination, and Vasco (SESVanderHave, Tienen, Blegium) for phenotyping plant disease dynamics. The seeds were sown 1*cm* deep in polypropylene pots filled with Fruhstorfer soil type P 25 (HAWITA Gruppe GmbH, Vechta, Germany). After seven days, 15 germinated seedlings of the variety BTS 8750 were individually transplanted into 1*l* round polypropylene pots filled with fertilized sandy topsoil (Gustav Lehmann Mörtel- u. Kieswerke GmbH, Burgdorf, Germany). The sugar beet plants were then cultivated for 66 days (14:10*h* light/dark photoperiod, 25.5${\pm }5.5^{\circ }C$, 55.4${\pm }$18${\%}$ relative humidity and about 225${\mu }mol/[sm]$ full spectrum light) in the greenhouse until they reached BBCH-Code 19 [32]. During cultivation, the plants were watered daily and fertilized every three weeks with 50*ml* of a 1:40 liquid fertilizer (liquid universal fertilizer, EDEKA, Hamburg, Germany) tab water mixture.

For the usecase data assessment 14 sugar beet plants of the variety Vasco were split into two variants: inoculated and non-inoculated. The inoculated variant was treated with liquid *Cercospora beticola* spore suspension produced from the strain 145 (BASF SE, Ludwigshafen, Germany) by spraying 7*ml* of the suspension with a concentration of ca. 30,000 spore per millilitre equally distributed on abaxial and adaxial side of the sugar beet leaves twice, in a time spawn of one hour. Afterwards the plants were covered with foil and the temperature in the greenhouse was increased to a minimum of $28^{\circ }C$ for six days. After six days the foil was released and the temperature was decreased. The sugar beets were then cultivated for the time of the data collection at 26.6${\pm }6.1^{\circ }C$, 55.2${\pm }$18.8${\%}$ relative humidity and ca. 225${\mu }mol/[sm]$ of full spectrum light.

### Measurements

#### Spatial quality assessment

The spatial resolution of the HC was tested using the international standard method called sine-wave based spatial frequency response (s-SFR) for measuring resolution and image sharpness of digital cameras [28]. Therefore, a sinusoidal Siemens star SFR chart with 144 cycles (ISO 12233:2023) [33] was measured with the HC at different working distances. Before measuring the SFR chart, the white reference was measured with the external illumination at a distance of 50*cm* and with an exposure time of 1000${\mu }s$ and a gain of 12. To replicate the referencing scheme of a plant measurement, the referencing measurement was done only once at the optimal working distance. The varying working distances are resembling the z-axis latitude of a plant canopy. The distance of the s-SFR chart to the lens was varied ${\pm }$ 5, 7.5 and 10*cm* from the optimal working distance of 50*cm* for the different measurements to simulate a plant canopy height of 20*cm*. The exposure time for the s-SFR chart measurements was set to 5000${\mu }s$ to increase the sensitivity of the HSI-system.

#### Spectral quality assessment

To validate the spectral accuracy of the HC, a calibration material (ColorChecker Classic, Calibrite LLC, Wilmington, USA) was measured with the same setup as described in section 2.2. The calibration material and the white reference was measured at the optimal working distance of 50*cm* with the external halogen illumination. In order to compare measurements with a high resolution non-imaging spectrometer (ASD Fieldspec Hi-Res, Malvern Instruments, Malvern, United Kingdom, software: $\hbox {RS}_{3}^{TM}$) the calibration material and the white reference were measured with the same exposure time (5000${\mu }s$) and gain (3.1) to enable comparison between the spectrometer and the HC. Prior to measurements, the spectrometer was warmed up for 30*min* and a contact probe was mounted. Reference measurements were made by placing the contact probe on the same white reference used for the HSI reference measurement. For the measurements of the calibration material with the spectrometer, the contact probe was pointed into the tiles of the calibration material and 10 repetitions of each tile were assessed.

#### Illumination quality assessment

The variability of the integrated high-power LED array of the HC was investigated for plant phenotyping routines and compared to external halogen illumination. For this purpose, sugar beet plants were measured with the HC illuminated by the integrated LED array and external halogen lamps respectively. Therefore, five sugar beet plants were placed in the center of the rectangle formed by the halogen illumination tripods in the measuring setup (Fig. 3) on the same position for individual measurements. The HC was leveled at a distance of 50*cm* to the approximate vertical center of the plant canopy. The distance between the halogen lamps and the canopy was about 75*cm*. Before measuring the plant material, the white reference was measured with the integrated and external illumination at a distance of 50*cm* and with an exposure of 1000${\mu }s$ and a gain of 12. The exposure time for the measurements was set to 5000${\mu }s$. Each plant was measured with the external and integrated illumination in exactly the same position, respectively.

Subsequently, the selected plants were measured with the non imaging spectrometer. Five leaves of each plant were randomly selected and the adaxial side of the leaves was measured in the middle next to the leaf vein with the leaf clip attachment of the spectrometers contact probe.

#### Time series measurement of disease dynamics

The optimal working distance of 50*cm* between the object and the lens was adjusted to the vertical center of the sugar beet canopy. The external halogen illumination setup was adjusted to account for the different heights of the sugar beet pots, resulting in different sugar beet canopy distance. The height of the halogen lamps was adjusted to 79*cm*, which resulted in a distance of approximately 60*cm* between the lamps and the sugar beet canopy and an angle of approximately $45^{\circ }$. Plants were measured with the HC one day before inoculation and 7, 11, 14, 21, 24 and 31 days after inoculation (dai). As reference data, disease severity was visually assessed as a percentage of infected leaf area via human expert rating [34].

### Data analysis

#### Spatial resolution

The acquired hyperspectral images for spatial quality assessment, considered as raw data, were processed in R (version: 4.3.1) [35] and RStudio (version: 2023.12.0+369, Posit PBC, 2023) using the package “hsdar” (version 1.0.4) [36]. First, the hyperspectral images of the plant material were normalized pixel by pixel with the corresponding illumination reference measurement using the following formula.

$$reflectance = \frac{HSI - sensor dark current }{reference HSI - sensor dark current}\times\frac{exposure\;reference}{exposure\;data}$$

Then RGB images of the normalized hyperspectral images were visualized (png, version: 0.1-8) [37]. The wavelengths chosen for RGB visualisation were 525*nm*, 550*nm* and 600*nm*. The RGB images of the different measuring distances were analyzed in the MathWorks tool IE-Resolution (Single Star Version, Image Engineering, Frechen, Germany) [38] to calculate the limiting resolution. The tool divides the sinusoidally modulated starburst pattern into 8 segments and calculates the Nyquist-frequency by a modulation transfer function (MTF) for each segment. In addition, the sum of the modulation for each distance and each segment was calculated to compare the sharpness of each segment within a working distance. The standard deviation of the modulation between segments was also calculated for each working distance.

#### Spectral accuracy

The hyperspectral images of the color calibration material measurements were normalized as described in section 2.5.1. Arrays of size 60*60*px* were extracted from different calibration material tiles of the hyperspectral images. From each array an average spectrum of the 10 measurement repetitions was calculated in R (asdreader, version: 0.1-3) [39]. For further analysis, only spectral bands measured by both sensors, the spectrometer (with a spectral resolution of 1*nm*) and the HC (spectral resolution: 2*nm*) were considered. A Pearson correlation was calculated between the measurements of each tile from the HC and the spectrometer using the “cor” function of the “stats” package (version: 4.3.1) [35]. Differences between the measurements from the HC and the spectrometer in reflectance of the considered spectral bands were calculated for each tile by subtracting the reference values of the spectrometer data from the HC data. For graphical presentation, of the results of this research the “ggplot2” package (version: 3.4.4) [40] was used.

#### Illumination comparison

The hyperspectral images of the illumination system comparison were normalized as described in section 2.5.1. The sugar beet canopies of the normalized data were segmented by creating two binary masks with the optimized soil adjusted vegetation index (OSAVI) and the difference vegetation index (DVI) [41, 42] with the following formulas.

$$OSAVI = 1.16\times\frac{800nm - 670nm }{800nm + 670m + 0.16}$$; $$DVI = 800nm-680nm$$

Both masks were merged to increase the segmentation performance using the “OR” operator [43]. After merging the masks, an image erosion was performed (mmand, version: 1.6.3) [44] to eliminate false segmented individual pixels. In addition, an average spectrum and the standard deviation of the average spectrum were calculated for each merged mask of the sugar beet canopy. Ten reference measurement repetitions of the non-imaging spectrometer per leaf, were averaged and the arithmetic mean of five leaf measurements per plant was calculated to estimate a representative spectrum for each of the five sugar beet canopies.

#### Image preprocessing and CNN training for CLS disease severity estimation

Plant masks were plotted for human annotation of the dataset which had been separated in training and test dataset for machine learning. After normalization as described in section 2.5.1, the first 2 spectral bands have been deleted due to sensor noise for further analysis. The Savitz-Golay smoothing filter [45] was applied to the hyperspectral images using the R package “gsignal” [46] (filterorder = 3, filterlength = 5). The smoothing filter was applied to reduce noise in the spectral data and to compute the first and second derivatives of the smoothed hyperspectral images. Pseudocolor images of the OSAVI index, as described in Section 2.5.3, were plotted with the smoothed, normalized data to improve the visibility of borders between different objects which supports human class annotation for machine learning. Three classes were defined for annotation of the pseudocolor images: “background”, “healthy” plant tissue, and CLS “diseased” plant tissue. The pseudocolor images of 2 inoculated and 2 non-inoculated sugar beet plants of each measurement date were randomly selected and the pixels of the images were annotated to the three defined classes using the software GIMP (version: 2.10.36, The GIMP Team). This resulted in the establishment of 3 separate masks of the defined classes. The training and test data sets were separated by measuring time points: 2 of the 8 time points (14 and 24 dai) were kept for the test dataset and the other 6 (0, 7, 11, 18, 21, 24 and 31 dai) for the training dataset. This division was made to establish two completely unknown disease progression states (early and late) in the time series for the CNN to test its performance. Which resulted in assigning 8 hyperspectral images to the test data and 24 hyperspectral images to the training dataset. The masks of the training dataset were used to generate matrices from the arrays of normalized and smoothed hyperspectral images by summarizing the reflectance values of the smoothed hypercube, the first and second derivatives for each pixel. The matrices of the training data set were then accumulated and outliers were removed (Rlof, version: 1.1.3) [47] by deleting the top 1$\%$ of the maximum and minimum values of each class. After removing the outliers, the three classes were balanced in terms of their total number of pixels to prevent the model from becoming biased towards one class. Since the total number of pixels of the diseased class is the lowest, 2 times the total number of pixels of the diseased class was set as the downsampling size for the other two classes.

The preprocessed training data set was then used to train a CNN specifically designed to analyze spectral data [30]. The implemented CNN focuses on analyzing and classifying the spectral data of each pixel. The input data were individual pixels with the corresponding spectral values combined from the smoothed spectrum and its first and second derivatives. The architecture of the CNN was adapted as described in [48] (retrieved: 2023.11.27). In addition, the batch size was set to 1024 pixels per epoch and the total number of epochs was set to 50. For the training and testing process, the smoothed hypercubes and the first and second derivatives were summarized for each pixel. 20$\%$ of the training data was retained to validate the training process. The training process was focused on the disease and background classes to better discriminate these classes the “classweight” function of the R package “keras” (version: 2.13.0) [49] was used. Furthermore, the adam optimizer with a learning rate of 0.001 was used for the training process. The trained model was then used to predict the affiliation of the pixels to three defined classes of the entire dataset which was centralized and scaled by the coefficient calculated in the training process. The trained model then predicted the affiliation of each pixel to the three defined classes from the summarized spectral values. With the predicted affiliation of each pixel, pseudocolor masks of each hyperspectral images were generated. To improve the performance of the CNN, post-processing steps were performed. First, the masks of the diseased and healthy classes were merged, then an image closing and erosion (mmand, version: 1.6.3) [44] was conducted to assign the mixed edge pixels to the class background and to assign single misaligned diseased pixels to the class “healthy”. A confusion matrix for the metrics precision, recall, specificity and F1 was calculated to analyze the performance of the disease severity estimation pipeline by comparing the predicted and post processed pixel class affiliation of the test data set compared to the human annotated masks.

With the final predicted and post-processed masks, the disease severity of each plant and each time point was calculated using the following formula which enumerates the leaf area affected by the disease [50].

$$disease\;severity=\frac{pixel\;count:\;diseased}{pixel\;count:\;healthy+pixel\;count:\;diseased}\times 100$$

In addition, the disease severity values of each time point from either the calculated CNN results or the visual assessment were checked for outliers by Dixon’s extreme value analysis [51] with a significance level of 5$\%$, which was performed in R using the outliers package (version: 0.15) [52]. A single outlier was detected in the CNN derived disease severity data set at 18 days after inoculation and removed for further analysis. To obtain comparable hyperspectral images, all plants were measured from the same perspective. Since plants grow differently even when grown under the same conditions, important parts of the plant may be missed in the measurements, leading to outlying measurement results. In addition, a Wilcoxon signed rank test [53] was performed in R with the “coin” package (version: 1.4-3) [54] to test for statistically significant differences between the disease severity of inoculated and non-inoculated variants for each measurement time point after 14 days post inoculation, which was the starting point for CLS symptom development. The mean disease severity for each variant at each time point and the standard deviation were calculated.

## Results

### Spatial resolution and image sharpness

The Nyquist frequency of the hyperspectral images measured at the different working distances with the HC between 40-60*cm* (Table 3) slightly increased with narrow working distances and slightly decreases with further working distances. The Nyquist frequency of the image recorded by the RGB sensor (1080) was exactly half the sampling rate of the RGB sensor (2160). The standard deviation between the segments of the HC increased as the working distance decreased. Furthermore, the HC presented differences between the segments by the summed modulation within a working distance, which allows to distinguish between sharpness of the different segments. Images measured in narrow and optimal working distances constitute mostly segment 5 as the sharpest and segment 7 as the most blurred segment. The results of the increased working distance identified segment 8 as the sharpest and segment 2 as the most blurred segment.

**Table 3:** Image sharpness of the HC at working distances between 40-60*cm*. SD shows the standard deviation between the summed modulation transfer function results for each segment

Distance[*cm*]	Nyquist frequency	Best segment ($\sum$MTF)	Worst segment ($\sum$MTF)	SD
40	239.5	5 (154.8)	7 (31.2)	43.4
42.5	239.5	5 (122.0)	7 (36.0)	28.3
45	238.5	1 (103.9)	7 (43.0)	19.7
50	238.5	5 (63.5)	7 (29.7)	12.5
55	237.5	8 (36.4)	2 (12.7)	9.0
57.5	235.5	8 (30.1)	2 (8.2)	8.3
60	228	8 (23.4)	2 (5.0)	7.5
50 (4K)	1080	3 (297.5)	5 (237.8)	19.4

### Spectral accuracy of the hyperspectral camera

**Fig. 4:**
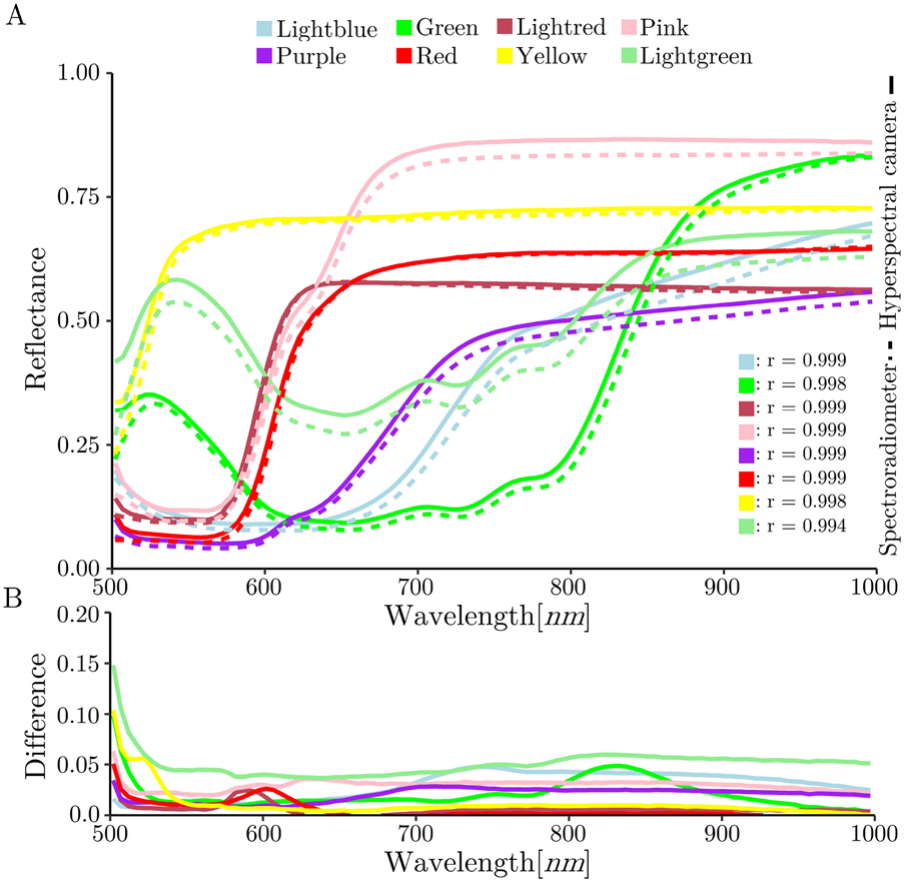
Spectral accuracy results. Spectral response of 8 tiles of the calibration material measured by the HC and the spectrometer are shown (**A**). The colors correspond to the colors of the measured calibration material tiles. “r” values present Pearson correlation coefficients between measurements from the spectrometer (reference) and the HC of the corresponding tiles. In addition, the differences in reflectance between the HC measurements and the spectrometer measurements are presented (**B**)

The displayed color calibration material measurements presented strong similarities between the HC and the reference measurements of each calibration material color tile with an r >0.99 for all the measured color tiles of the calibration material. In addition, the differences between the HC and spectrometer measurements were all less than 0.1 reflectance between 512-1000*nm* (Fig. 4B).

### Illumination system quality

**Fig. 5:**
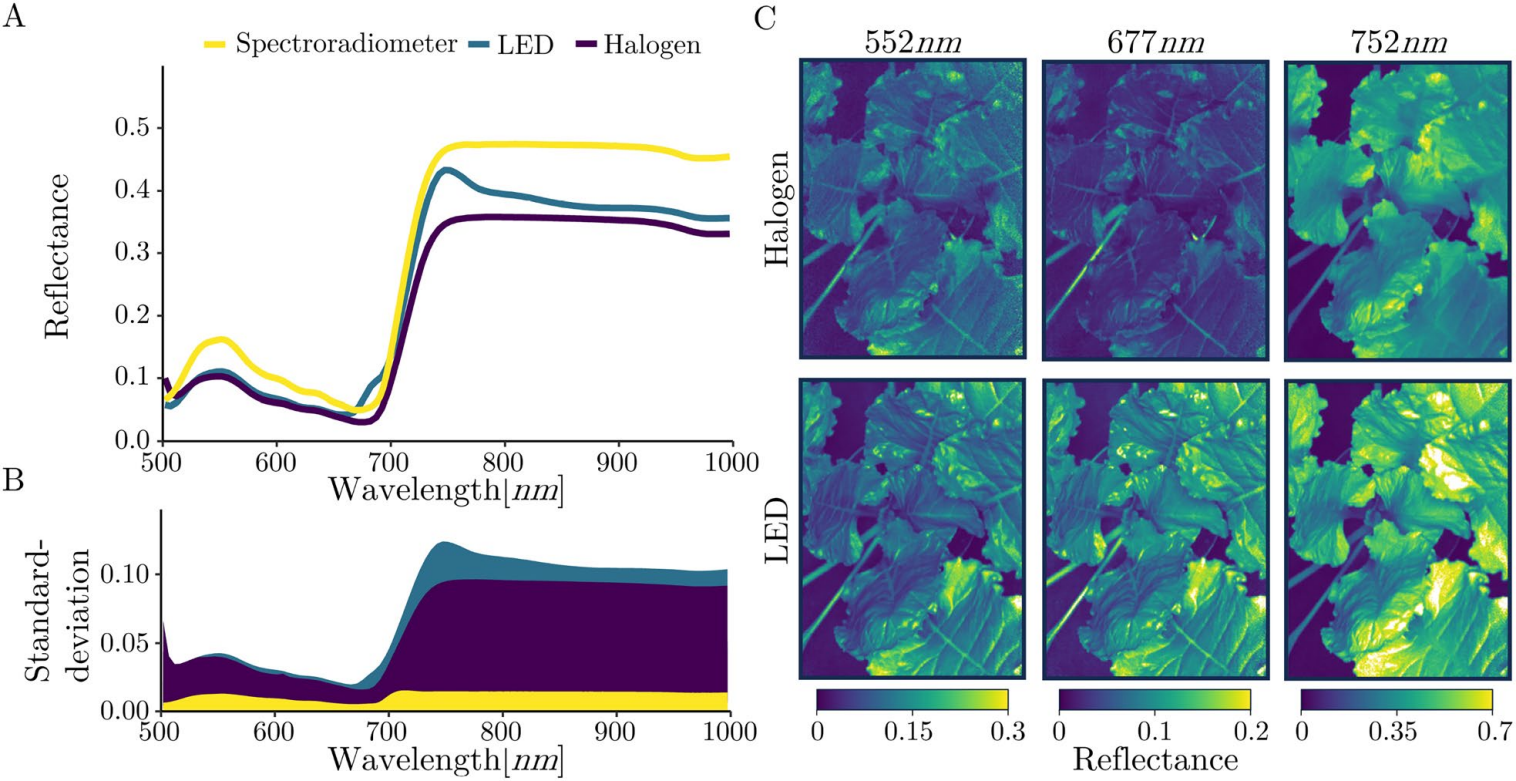
Comparison of illumination systems by sugar beet canopy spectral responses. The arithmetic mean of the five averaged canopy reflections between 500 and 1000*nm* of the two different illumination systems and the spectrometer measurement is shown (**A**). In addition, the arithmetic mean of the standard deviations of the reflectance of the averaged canopy masks and the averaged spectrometer reference measurements is displayed (**B**). Furthermore, the variance of the measured reflectance over a plant canopy of the spectral bands 552*nm*, 677*nm* and 752*nm* is presented (**C**)

Differences in the arithmetic mean of reflectance values were identified around 677*nm* in the VIS and around 752*nm* in the NIR. (Fig. 5A). The spectral curve of the spectrometer reference measurement corresponds to the curve of the halogen-illuminated measurements. However, the reference curve has an increased offset of approximately 30$\%$ compared to the halogen-illuminated curve. The LED illumination caused high standard deviation at 525*nm* and 1000*nm*, which reaches more than 0.1 reflectance at approx. 720*nm* (Fig. 5B). The spectrometer measurements constantly had the lowest standard deviation over the entire measured spectrum. Furthermore, the LED illumination produces the highest spatial reflectance variance over the sugar beet canopy in all three spectral bands (Fig. 5C) compared to the halogen illumination. In particular, the spectral band at 752*nm* of the LED illumination hyperspectral images includes pixels located at the highest leaf base with reflectance values that are off scale and therefore displayed in white.

### Cercospora leaf spot disease severity prediction

**Fig. 6:**
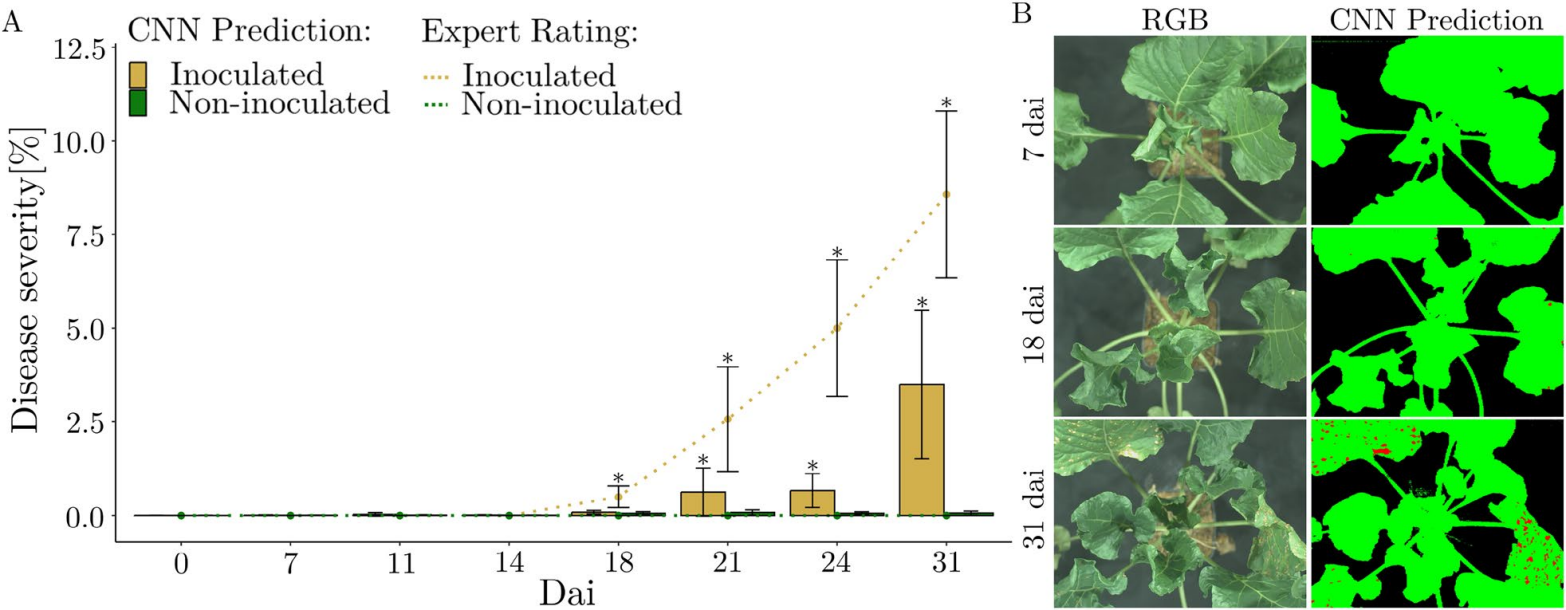
Disease severity results from CNN and expert scoring. Bars show disease severity from the CNN pipeline and dotted lines show the expert assessment. Error bars show ± standard deviation and stars show statistically significant difference between variants at a given time point at a significance level of p<0.05 (**A**). RGB images from the 4K sensor of the HC and colored masks from the CNN pipeline of the three classes background (black), healthy (green), disease (red) at three selected measurement times (early, intermediate, late) of a replicate of the inoculated variant are shown (**B**)

The CNN and the human expert detected the first symptoms of CLS on the inoculated plants 18 days after inoculation (Fig. 6A). During the following seven days, the mean disease severity increased by 4.5$\%$ according to the expert rating and 0.57$\%$ according to the CNN. At the final day of measurement, the disease severity was 8.57$\%$ for the expert rating and 3.49$\%$ for the CNN pipeline. The disease severity rating by the expert for the non-inoculated variant was zero percent throughout the observation period. The CNN pipeline showed a low false positive rate for disease severity results of the non-inoculated variant, with a maximum of 0.081$\%$. The standard deviation increased for both assessment methods as the CLS infection progresses. The inoculated and non-inoculated variants showed statistically significant differences for both rating methods at 21, 24, and 31 days after inoculation. The CNN pipeline pixel affiliations of one repetition of the inoculated class to the RGB images of the same plant at the respective time points are compared (Fig. 6B). The RGB and pseudo color images of the CNN pipeline display comparable infection patterns at all time points upon visual analysis.

The CNN has a precision of over 98$\%$ for the background and healthy classes. For the diseased class, the precision is approximately 62$\%$ (Table 4). The false positive rate of pixel affiliations in falsely predicted classes is consistently below 5$\%$ for every class combination except when healthy is predicted but truly diseased, which has a false negative rate of 33$\%$ (Table A1). The CNN consistently achieves over 98$\%$ in classifying healthy and background pixels in terms of recall, specificity, and F1 score. In the classification of diseased pixels, the specificity is above 99$\%$ and the precision, recall and F1 score are above 60$\%$.

**Table 4:** CNN performance matrix of the pixel wise classification from the test dataset

	Precision	Recall	Specificity	F1
Background	0.98	0.98	0.98	0.98
Diseased	0.62	0.60	0.99	0.61
Healthy	0.98	0.98	0.98	0.99

## Discussion

### Hyperspectral imaging quality assurance for plant phenotyping

Accurate imaging of three-dimensional (3D) objects such as plants is challenging, especially in the field of HSI. Every image has the phenomenon of edge pixels, which contain a mixed spectral signature of the overlapping objects. In the case of plant phenotyping, for example, this can occur at the boundary between leaves and background or healthy leaf tissue and diseased leaf tissue. In conclusion, high image resolution resulting in a small ground sampling distance (GSD) is advantageous for segmenting objects in a hyperspectral image. However, this requires an adequate image sharpness. The Nyquist frequency takes the image sharpness into account and describes the resolution limit of an image and therefore also the resolution limit of an imaging system [28]. Apart from the technical limitation of the system, the GSD can only be influenced by the measuring altitude. The image sharpness, on the other hand, is mainly influenced by the focal ratio of the camera, which is fixed for the HC and determines an optimal working distance of 50*cm*, resulting in a GSD of 0.2*mm* [55]. Even though an optimal working distance has been determined, changing the distance may be necessary in certain scenarios, such as in including the entire plant in the hyperspectral image or in reducing the GSD to measure small objects.

The critical sample value of half of the maximum image pixel height (240) was narrowly missed by the HC at any working distance, resulting in a small aliasing and loss of information [56]. By changing the working distance ${\pm }$10*cm* the GSD changed ${\pm }$0.04*mm* which can be the reason for the slight variation in the Nyquist frequency. The technical aspects of the HC concerning the low focal ratio would not assume any remarkable changes in the image resolution between at least ${\pm }$5*cm* from the optimal working distance which is corresponding with the image resolution test results. Low focal ratios are associated with a wide image focus which is beneficial for measuring 3 dimensional objects. The differences in sharpness between the segments within a working distance could have been caused by faulty technical components of the HC. Defects related to the hardware of the sensor chip would have resulted in a locally uniform degradation of quality at any working distance. Since the blur of the segments varies over the working distances, defects in the installed lens may have caused this problem. Point defects on the lens can occur during the manufacturing process, such as bubbles and uneven coating, and during the manufacturing and assembly of the camera [57]. The position of the sinusoidal Siemens star chart under the lens of the HC was automatically corrected by the MathWorks tool IE-Resolution. Shifting the angle between the chart and the lens when the working distance was adjusted might have caused the shifting of the weak spot concerning the sharpness. In addition to the technical aspects of the camera, the resulting image resolution and sharpness is affected by the signal processing within the system. The actual sensor resolution of the HC is 1920*1080*px*. The stored hyperspectral image only has a resolution of 640*480*px*, which means that spatial binning is applied to probably increase the spectral signal-to-noise ratio [58].

In summary, the international standard method for the evaluation of image sharpness of digital cameras [28] can be considered to be applicable to HSI-systems. Since the method evaluates contrast, simple black and white images can also be evaluated, allowing the analysis and comparison of the image sharpness of each spectral band measured by an HSI-system, if needed.

To investigate the spectral accuracy of the HC, the spectral measurements of a calibration material for digital cameras were compared to the spectral measurements of the same calibration material of a high spectral resolution non-imaging spectrometer. The average Pearson correlation between the measurements of the 8 tiles of the calibration material resulted in a correlation of about 99.8$\%$, certifying the installed spectral sensor in the HC a high accuracy. The reflectance differences of all measured tiles between 512-1000*nm* are below 0.1, which corresponds to a high average correlation. However, between 500-512*nm*, the differences increase due to the low signal-to-noise ratio of the first measured bands of the HC. This is a common problem in spectroscopy, often resulting in the elimination of the first noisy spectral bands for analysis. The low irradiance of the halogen illumination in the blue light region commonly used for HSI exacerbates the effect, as shown by the distortion of the spectrum in the affected spectral region [25]. The slight spectral shift between the HC and the reference measurements which occurs for most of curves may be caused by technical differences in the spectral measurements or by different processing of the measured signals. In general, comparing the spectral measurements of a calibration material’s spectral response with those of a reliable spectrometer is an easy-to-apply method for evaluating the accuracy of an HSI-system.

### Illumination for hyperspectral imaging

The accuracy of HSI is heavily influenced by the light conditions during measurements [25, 59]. Stable and sufficient illumination is crucial for generating high-quality data in laboratory setups under controlled conditions. In-field measurements are the most reliable source for extracting plant traits because environmental influences are considered. Generating high-quality and accurate hyperspectral images in the field is challenging due to unpredictable and unstable natural light conditions. To stabilize the light conditions for hyperspectral measurements, the tested HC supports illumination by an integrated light source. The integrated LED light of the HC was evaluated by comparing measurements of sugar beet plants in controlled conditions with common halogen-illuminated and non-imaging spectrometer measurements.

When comparing the courses of the spectral curves, it is noticeable that the spectrometer and halogen-illuminated measurements exhibit similar trends. However, there is a significant shift between these curves, which can be attributed to the 3D structure of the plant. The spectrometer measurements were conducted using a plant probe with a leaf clip attachment that flattened the leaf for the measurement, resulting in a two-dimensional equally illuminated measurement. Creating equally distributed illumination on a 3D structure is challenging. Additionally, combining this with a two-dimensional normalization for hyperspectral measurements, which is still the state of the art in the field of plant phenotyping [12, 14, 60, 61], shifting of the spectral response can occur. Research has been conducted to correct the calibration of HSI by taking into account the plant geometry [12] and shadowing effects [62]. Though, both approaches do not take into consideration the light distribution of the illumination source in the set up. Another approach to correct this issue would be to measure the calibration material at different altitudes of the z-axis and correspond this to the depths of the hyperspectral image in the normalization process. This can already be achieved with tools like Depth Anything [63].

Between 650-775*nm*, the spectral curves of halogen and LED illumination significantly diverge, exhibiting two abnormal peaks in the LED illumination measurements at approximately 677*nm* and 752*nm*. Additionally, certain hotspots exceeding a reflectance value of 1.0 are visible in the reflectance heat maps of LED-illuminated hyperspectral images. These abnormal spectral responses may have resulted from mirroring effects caused by the non-diffuse integrated LED light which incidents at a $90^{\circ }$ angle. To prevent mirroring effects, the halogen illumination was mounted at an angle of approximately $45^{\circ }$ to the plant, and diffusing screens were attached. The lambertian property of leaves is generally dependent on surface characteristics, particularly the amount of epicuticular wax, which can enhance mirroring effects [64]. In principle, LEDs as an illumination source for HSI imply a lot of advantages compared to halogen illumination such as cost effectiveness, longer lifetime, less heat emission and increased irradiance in the blue light region [24, 25, 65]. However, the comparison of illumination sources in this research highlights the importance of evenly distributed diffuse illumination for HSI.

### Proof of concept: Neural network supported CLS disease severity estimation

In addition to the HSI-system quality evaluation pipeline, this research proposes a concept for a CNN multiclass classification of quality assured hyperspectral images in the context of phytopathology. A CNN specifically designed for the analysis of spectral data [30] was trained for the detection of CLS diseased pixels. The trained CNN was used to estimate the disease severity of CLS-infected plants in a time series and compared with visual expert assessments. The CNN underestimated the disease severity over the whole time series compared to the expert rating. However, the use of human expert ratings as reference data has been widely discussed in the literature for the last 100 years [66]. To establish a true image-based reference dataset, the entire dataset must be classified by human annotation. With the increasing amount of data in high throughput plant phenotyping, this approach is impractical. The performance of the trained CNN for multiclass classification shows high precision, recall, specificity and F1 for the healthy and background classes, which means the CNN is very accurate in segmenting the vegetation from the background in a hyperspectral image. In addition, the diseased class has a high specificity, which describes the relationship between the true negative and all negative pixels of this class. Precision, recall and F1 for the diseased class are above 0.6, which is a positive result for disease classification. The presented CNN performance for the classification of the diseased class explains the possible underestimation of the CLS disease severity. The early symptoms of CLS are small reddish-brown lesions with a white to grayish center in which black pseudostroma are formed. As the infection progresses, the lesions grow and coalesce, which can lead to a complete collapse of the leaf [21]. Detecting early symptoms using a pixel-by-pixel approach is difficult as a single lesion at this stage may contain more edge pixels with mixed spectral responses than clear central lesion pixels. In addition, CLS induced non-visible spectral changes may occur that are not considered in human annotation and are also overrepresented in early stages of infection. Possible non-visible symptoms around the lesions prohibited human-driven post-processing steps such as mask closure applied to the diseased class. An increased performance of the CNN on more advanced infection stages is expected. In addition, an increased number of repetitions, apart from a proof-of-concept trial, which consequently enlarges the training dataset, will result in a reduced standard deviation of the disease severity estimation and presumably an improvement of the CNN performance as well. Additionally, the not adjustable spatial binning of the HC reduced the resolution from 1920*1080*px* to 640*480*px* of the hyperspectral image which consequently reduces possible precision of localizing symptoms in the spatial dimension. Furthermore, changing the pixel-by-pixel disease classification approach to a semantic segmentation approach by using a mask r-cnn or a U-net [67, 68] would focus the analysis on the spatial arrangement of pixels. The benefits of focusing the analysis on the spatial rather than the spectral information need to be tested. In summary, the proposed concept consists of a reliable pipeline for estimating the disease severity of CLS based on quality assured hyperspectral images under controlled conditions.

## Conclusion

A neural network supported analysis concept was successfully applied to quality assured hyperspectral imaging data for a phytopathological usecase. Furthermore, the assessment of spatial and spectral accuracy of an HSI-system by the proposed pipeline supports decision making for the selection of the right HSI-system for the required application. Furthermore, internal signal processing, such as spatial or spectral binning, can be evaluated and adjusted for the desired hyperspectral image quality. Illumination for HSI in coherence with real radiometric correction rather than simple normalization is still complex, especially for the measurement of 3D objects. The results confirm the importance of diffuse and uniform illumination for HSI. If the results of the spectral accuracy assessment and the illumination testing are compared, the effect on the spectral response of measuring 3-dimensional objects which are based on two-dimensional normalization is presented. For physically accurate radiometric correction of 3D measured objects the light distribution in the z-axis must be considered. Quality assured hyperspectral images can reduce limitations in the data analysis and provide high quality data sets for plant phenotyping. The presented concept for CNN supported CLS disease severity estimation based on hyperspectral images was successfully implemented. Furthermore, reducing spatial binning for pixel-by-pixel classifications, or switching the approach to a semantic segmentation classifications by using a Mask R-CNN or U-Net instead of a 1-dimensional spectral response focused CNN can enhance the performance.

In conclusion, the quality assurance pipeline is independent concerning the type of the HSI-system because it is data based, analysing the resulting hyperspectral image. Therefore, it can be easily applied on any other HSI-system to assess and assure the presented quality parameters.

## Data Availability

Upon request.

## Appendix A

See Table 5

**Table 5:** CNN confusion matrix of the pixel wise classification from the test dataset

Prediction/Target	Background	Diseased	Healthy
Background	1148565	152	22040
Diseased	41	1985	1272
Healthy	13861	1055	1268629
